# Should lymphadenectomy performed routinely in patients with primary intrahepatic cholangiocarcinoma undergoing curative hepatectomy? A retrospective cohort study with propensity-score matching analysis

**DOI:** 10.1186/s12893-023-02255-5

**Published:** 2023-11-30

**Authors:** Shan Huang, Jiu-Lin Song, Bo Li, Peng-Sheng Yi, Jian Yang

**Affiliations:** 1 Department of Nephrology, Sichuan Academy of Medical Science and Sichuan Provincial People’s Hospital, Sichuan Province, Chengdu, 610072 China; 2 Department of Pediatric Surgery, West China Hospital of Sichuan University, Sichuan Province, Chengdu, 610041 China; 3 Department of Liver Transplantation Center, West China Hospital of Sichuan University, Sichuan Province, Chengdu, 610041 China; 4 Department of Vascular Surgery, West China Hospital of Sichuan University, Sichuan Province, Chengdu, 610041 China; 5 Affiliated Hospital of North Sichuan Medical College, Sichuan Province, the Nanchong City, China

**Keywords:** Hepatectomy, Intrahepatic cholangiocarcinoma, Lymphadenectomy, Propensity score matching analysis

## Abstract

**Background:**

The benefit of routine lymphadenectomy (LD) in improving outcomes for patients with primary intrahepatic cholangiocarcinoma (ICC) undergoing curative hepatectomy remains unclear.

**Materials and methods:**

This study enrolled 269 consecutive patients who underwent liver resection for primary ICC from January 2009 to July 2020 in West China Hospital. The association of the nodal status with disease-free survival (DFS) and overall survival (OS) was analyzed using the Cox proportional hazards model and 1:1 propensity score matching (PSM) analysis.

**Results:**

Seventy-five (27.9%) patients underwent curative liver resection combined with LD (LD+ group), while 194 (72.1%) patients received curative liver resection without LD (LD- group and Nx group). Among the LD+ group, metastatic disease was present in 36 patients (48%, N1 group) and absent in 39 patients (N0 group). During the follow-up period, 116 patients (43.1%) experienced tumor recurrence and 101 patients (37.5%) died due to recurrence. Multivariate analysis revealed that lymph node metastasis (N1, HR 3.682, 95% CI 1.949–6.957, *p* < 0.001) was associated with worse OS, while LD+ status (HR 0.504, 95% CI 0.298–0.853, *p* = 0.011) was associated with improved OS. Adjuvant therapy was a protective factor for both DFS (HR 0.602, 95% CI, 0.447–0.810, *p* = 0.001) and OS (HR 0.683, 95% CI 0.484–0.963, *p* = 0.030). After 1:1 PSM, the LD+ patients (*n* = 74) displayed similar 1-, 3- and 5-year DFS rates (40.0, 7.9 and 7.9% vs. 29.0, 13.7 and 13.7%, *p* = 0.741) and OS rates (56.0, 26.6 and 22.2% vs. 58.9, 25.6, and 16.4%, *p* = 0.644) to the LD- patients (*n* = 74). Additionally, among the 75 LD+ patients, 48 patients underwent hepatic hilar lymphadenectomy (HHL), and 27 patients underwent extended hepatic hilar lymphadenectomy (EHL). Both DFS (*p* = 0.504) and OS (*p* = 0.215) were similar between the HHL and EHL groups.

**Conclusion:**

Routine LD and adjuvant therapy may contribute to improved OS according to the crude analysis. LD could provide accurate staging without excessive risk and guide adjuvant therapy based on the tumor stage, potentially resulting in better survival. These results suggest that a routine LD should be considered during curative hepatectomy for ICC.

**Supplementary Information:**

The online version contains supplementary material available at 10.1186/s12893-023-02255-5.

## Introduction

Intrahepatic cholangiocarcinoma (ICC) originates in the hepatic parenchyma either directly from cholangiocytes or from the transdifferentiation of hepatocytes. In recent decades, the incidence of ICC has dramatically increased, making it the second most common primary hepatic cancer [1, 2]. Although hepatic resection remains the first-line curative treatment, the 5-year overall survival (OS) rates of ICC patients range from 30 to 35% after hepatectomy [3]. Recently, several studies have reported that the lymph node status is one of the most important prognostic factors for ICC [4–8]. Clinicians have focused on lymph node dissection in ICC patients, but its effects in ICC surgical radical treatment remain controversial [3, 4, 7, 9]. The true benefit of lymph node clearance for survival is unclear. Thus, we aimed to explore the impact of lymphadenectomy (LD) on ICC patients undergoing hepatectomy.

## Materials and methods

### Study population

Clinical data from all consecutive patients who underwent liver resection for primary ICC lesions at the West China Hospital of Sichuan University, China, from January 2009 to July 2020 were retrieved from a prospective database for this study. Inclusion criteria encompassed age > 18 years old, initial curative hepatectomy, and histological diagnosis with primary ICC lesions. Exclusion criteria involved age < 18 years old, presence of metastatic lesions at diagnosis (American Joint Committee on Cancer [AJCC] stage M 1[10]), palliative resection or treatment with non-surgical regimens, recurrent ICC lesions, and mixed ICC and hepatocellular carcinoma lesions.

### Data collection

Baseline data, including age and sex, hepatitis B virus surface antigen (HBsAg) status, Child–Pugh classification, aspartate aminotransferase (AST) level, carbohydrate antigen 19–9 (CA19–9) level, blood loss volume, intraoperative blood transfusions, perioperative complications, and tumor characteristics were collected for each patient. Specifically, these data included the presence of liver cirrhosis, tumor tumor size, number of lesions, morphological subtype, histological grade, and tumor invasion status (vascular/perineural/biliary). In addition, information regarding the surgical procedure (major or minor hepatectomy), treatment with LD, and adjuvant therapy (e.g. adjuvant chemotherapy, transcatheter arterial chemoembolization, and radiotherapy) were collected. The surgical margin and nodal status were determined from the final postoperative pathological report.

### Surgical techniques

Indications for lymphadenectomy during radical hepatic resection of ICC referred to preoperative suspicion on CT or MRI imaging or dubious intraoperative findings by surgical exploration [11]. Hepatic hilar lymphadenectomy (HHL) was performed as harvesting nodes in hepatoduodenal ligament (within station N0.12) [6]. Extended hepatic hilar lymphadenectomy (EHL) was defined as harvesting nodes beyond station No.12, extending to common hepatic artery, celiac artery, hepatogastric ligment, and/or peripancreatic area [6]. Major liver resection was performed as resection of three or more liver segments, according to intrahepatic lesion sizes and locations [12].

### Follow-up

After discharge, the patients were followed every 3 months during the first 2 years after the initial operation and every 6 months thereafter. Recurrence was defined as the appearance of a new lesion that exhibiting radiological features intra- or extrahepatically. The date of the last follow-up and the survival status were collected for all patients.

### Clinical terms

Tumor size was defined as the largest diameter (in centimeters) of the resected specimen. Further, histological grade was defined as moderately or poorly differentiated, and the highest histological grade was used to define the tumor grade in patients with multiple resected specimens. Postoperative complications were stratified according to the Clavien–Dindo classification [13]. Major complications were defined as those of grade IIIa or above. DFS was defined as the interval between the date of the operation and the date of the first recurrence diagnosis of the or the last follow-up. OS was measured from the date of the operation to the date of death or the last follow-up.

### Statistical analyses

Continuous data are expressed as the median and range/interquartile range (IQR). Qualitative variables are expressed as the frequency (percentage). Student’s t test or the Mann–Whitney U test was used for intergroup comparisons of quantitative variables as appropriate, whereas the χ2 test or Fisher’s exact test was used to compare categorical data. Survival analysis was conducted using the Kaplan–Meier method, and the significance of differences between survival curves was determined using the log-rank test. Multivariate regression analysis of the survival distributions was carried out using the Cox proportional hazard model. All of the variables included into the multivariate analysis were risk factors proven by previous researches for ICC, or with a *P* value less than 0.1 in univariate analysis. Statistical analyses were performed using SPSS 23.0 (SPSS, Inc., Chicago, IL). Two-sided *P* values < 0.05 were considered statistically significant.

To eliminate selection bias, patients were matched 1:1 by propensity scores [14] based on their baseline characteristics using the matching package in R software (version 3.1.0 for Windows, Bell Laboratories) and conducted with the 1:1 nearest neighbor matching method. After all propensity scores matching (PSM) was complete, we compared all baseline characteristics between different groups.

## Results

### Perioperative clinical characteristics

Patients were followed until the date of death or the final date of the study, December 30, 2020. A total of 269 patients underwent initial hepatectomy for primary ICC after the exclusion of 46 cases: presenting with metastatic disease at the time of diagnosis (AJCC stage M 1[10]) (*n* = 13); palliative resection or treatment with nonsurgical regimens (*n* = 9); recurrent ICC lesions(*n* = 7); mixed ICC and hepatocellular carcinoma lesions(*n* = 17). The majority of patients were male (*n* = 169, 62.8%), and most patients were younger than 65 years old (*n* = 215, 79.9%). Nearly two-thirds (*n* = 159, 60.2%) of the patients presented with a unifocal lesion. Only one in three patients presented with a tumor size < 5 cm (*n* = 89, 34.0%). More than half of the patients were positive for HBsAg (*n* = 148, 55.0%) and presented with cirrhosis (*n* = 146, 54.3%). The invasion of adjacent structures was not noted in most patients. Specifically, macrovascular invasion, microvascular invasion, periductal invasion, and perineural invasion were observed in only 12.6% (*n* = 34), 18.4% (*n* = 49), 5.9% (*n* = 16) and 7.5% (*n* = 20) of tumors, respectively. Poorly differentiated lesions were found in one-third (*n* = 83, 30.9%) of patients, and a positive surgical margin was found in 17 (6.3%) patients. Regarding liver function, as defined by the Child–Pugh classification, 254 (94.4%) patients were class A, 15 (5.6%) patients were class B, and none were class C.

Of the 269 patients, 171 (63.6%) underwent major liver resection, and 98 (36.4%) underwent minor resection. Complications occurred in 18 patients, including MODS in one patient, acute respiratory and circulatory failure in two patients, liver failure in three patients, respiratory failure in four patients, pleural effusion in four patients, intraabdominal bleeding in one patient, liver abscess in one patient, pulmonary embolism in one patient, and pleuro-peritoneal infectious shock in one patient. Three of these 18 patients died, and the remaining patients were cured. For histological examination, the mass-forming morphological tumor variant was most common (*n* = 263, 97.8%), followed by the papillary morphology (*n* = 4, 1.5%) and mixed mass-forming and periductal-infiltrating variant (*n* = 2, 0.7%). Of note, 75 patients (27.9%) underwent concomitant LD (LD+ group). Metastatic nodal disease was observed in 48% (*n* = 36) of these patients, who were therefore classified as N1 according to the AJCC TNM staging system [10]. The remaining patients without metastatic nodal disease were classified as N0, and the 194 patients who did not undergo concomitant LD were classified as Nx/LD-. Postoperatively, 70 (26.0%) patients received antiviral therapy, and 97 (36.1%) patients received adjuvant therapy, including adjuvant chemotherapy, transcatheter arterial chemoembolization and radiotherapy.

### Factors associated with DFS and OS

**Fig. 1:**
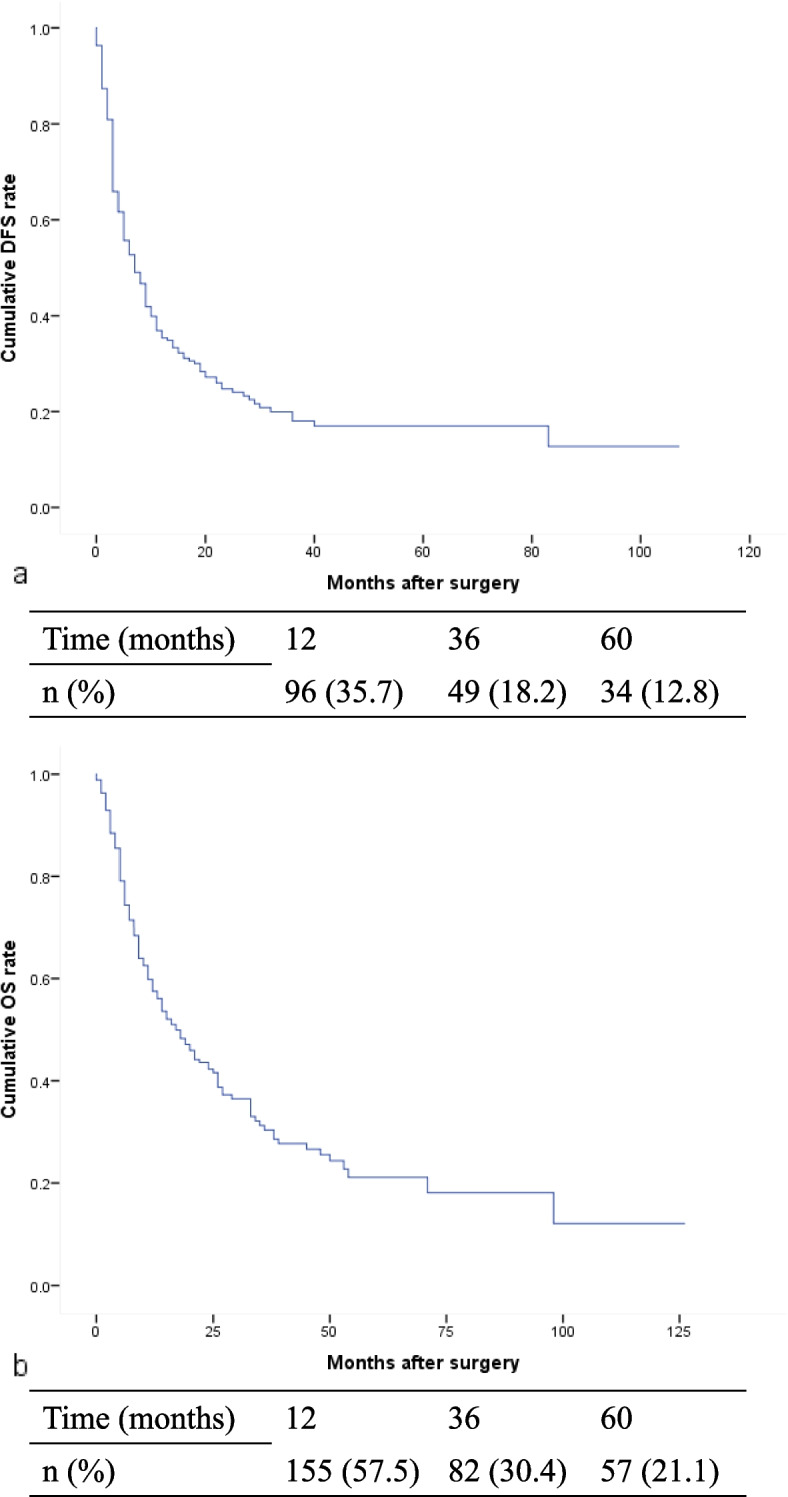
DFS rate (**a**) and OS rate (**b**) of in 269 ICC patients after surgery

The median follow-up duration was 17 months (1–125 months). During the follow-up period, tumor recurrence developed in 116 patients (43.1%) with a median DFS of 5 months (IQR: 3–13), and 101 patients (37.5%) died with a median OS of 11 months (IQR: 5–24). Specifically, the 1-, 3- and 5-year DFS rates were 35.7, 18.2 and 12.8%, respectively (Fig. 1a). While OS was 57.5% at 1 year after surgery, a dramatic decrease in survival was observed over time, as only 30.4 and 21.1% of patients survived for 3 and 5 years, respectively (Fig. 1b). Furthermore, non-tumor-related death occurred in 20 cases during the follow-up period: intracerebral bleeding in 2 cases; multiple organ dysfunction syndrome (MODS) in 2 cases; pulmonary failure in 3 cases; kidney failure in 1 case; gastrointestinal bleeding in 1 case; acute cardiac infarction due to atrial myxoma in 1 case; liver failure in 9 cases; and car accident in 1 case.

Multivariate analysis showed that AST ≥ 31 IU/L (HR 1.615, 95% CI, 1.192–2.188, *p* = 0.002), tumor diameter ≥ 5 cm (HR 1.679, 95% CI, 1.211–2.327, *p* = 0.002), multifocal lesions (HR 1.514, 95% CI, 1.120–2.048, *p* = 0.007), macrovascular invasion (HR 1.564, 95% CI, 1.034–2.364, *p* = 0.034), perineural invasion (HR 1.793, 95% CI, 1.004–3.204, *p* = 0.048), and periductal invasion (HR 2.127, 95% CI, 1.041–4.347, *p* = 0.038) were associated with reduced DFS. Notably, adjuvant therapy (HR 0.602, 95% CI, 0.447–0.810, *p* = 0.001) was an independent protective factor for DFS (Table 1). Moreover, reduced OS was noted among patients presenting with tumors ≥5 cm in diameter (HR 1.849, 95% CI 1.260–2.713, *p* = 0.002), multifocal lesions (HR 1.500, 95% CI 1.067–2.108, *p* = 0.02), macrovascular invasion (HR 1.569, 95% CI 1.038–2.370, *p* = 0.032), periductal invasion (HR 2.899, 95% CI 1.154–7.281, *p* = 0.024), CA19–9 ≥ 36.2 U/mL (HR 1.571, 95% CI, 1.119–2.207, *p* = 0.009), a positive margin status (HR 2.142, 95% CI 1.030–4.454, *p* = 0.042), and lymph node metastasis (HR 3.682, 95% CI 1.949–6.957, *p* < 0.001). Notably, adjuvant therapy (HR 0.683, 95% CI 0.484–0.963, *p* = 0.030) and lymphadenectomy (HR 0.504, 95% CI 0.298–0.853, *p* = 0.011) were protective factors for OS (Table 2).

**Table 1:** Clinicopathological factors associated with DFS in ICC patients

Variables	Univariate analysis	Multivariate analysis	
	*P* value	HR (95% CI)	*P* value
Age (≧65)	0.645		0.585
Gender (male)	0.007		0.225
HBsAg positive	0.006		0.418
Child-Pugh Class (A/B)	0.910		0.875
AST≧31 IU/L	< 0.001	1.615 (1.192–2.188)	0.002
CA-199≧36.2 U/mL	0.352		0.324
Tumor diameter ≧5 cm	< 0.001	1.679 (1.211–2.327)	0.002
Multifocal lesions	< 0.001	1.514 (1.120–2.048)	0.007
Major hepatectomy	0.175		0.482
Lymphadenectomy	0.304		0.920
Tumor differentiation (Poor)	0.004		0.122
Macrovascular invasion	< 0.001	1.564 (1.034–2.364)	0.034
Microvascular invasion	< 0.001		0.063
Perineural invasion	0.205	1.793 (1.004–3.204)	0.048
Periductal invasion	0.277	2.127 (1.041–4.347)	0.038
Margin positive	0.671		0.663
Lymph node metastasis	0.024		0.143
Pathological cirrhosis	0.010		0.349
Antiviral therapy	0.083		0.631
Adjuvant therapy	< 0.001	0.602 (0.447–0.810)	0.001

*AST* alanine aminotransferase, *CA19–9* carbohydrate antigen 19–9, *DFS* disease-free survival, *HBsAg* hepatitis B virus surface antigen, *HR* hazard ratio, *CI* confidence interval, *ICC* intrahepatic cholangiocarcinoma

**Table 2:** Clinicopathological factors associated with OS in ICC patients

Variables	Univariate analysis	Multivariate analysis	
	*P* value	HR (95% CI)	*P* value
Age (≧65)	0.304		0.219
Gender (male)	0.441		0.398
HBsAg positive	0.112		0.098
Child-Pugh Class (A/B)	0.821		0.809
AST≧31 IU/L	< 0.001		0.055
CA-199≧36.2 U/mL	0.001	1.571 (1.119–2.207)	0.009
Tumor diameter ≧5 cm	< 0.001	1.849 (1.260–2.713)	0.002
Multifocal lesions	< 0.001	1.500 (1.067–2.108)	0.020
Major hepatectomy	0.389		0.989
Lymphadenectomy	0.432	0.504 (0.298–0.853)	0.011
Tumor differentiation (Poor)	0.003		0.226
Macrovascular invasion	0.002		0.052
Microvascular invasion	< 0.001	1.569 (1.038–2.370)	0.032
Perineural invasion	0.004		0.056
Periductal invasion	0.071	2.899 (1.154–7.281)	0.024
Margin positive	0.202	2.142 (1.030–4.454)	0.042
Lymph node metastasis	< 0.001	3.682 (1.949–6.957)	< 0.001
Pathological cirrhosis	0.06		0.072
Antiviral therapy	0474		0.869
Adjuvant therapy	0.345	0.683 (0.484–0.963)	0.030

*AST* alanine aminotransferase, *CA19–9* carbohydrate antigen 19–9, *OS* overall survival, *HBsAg* hepatitis B virus surface antigen, *HR* harzad ratio, *CI* confidence interval, *ICC* intrahepatic cholangiocarcinoma

### Survival analysis after propensity score-matching analysis

**Fig. 2:**
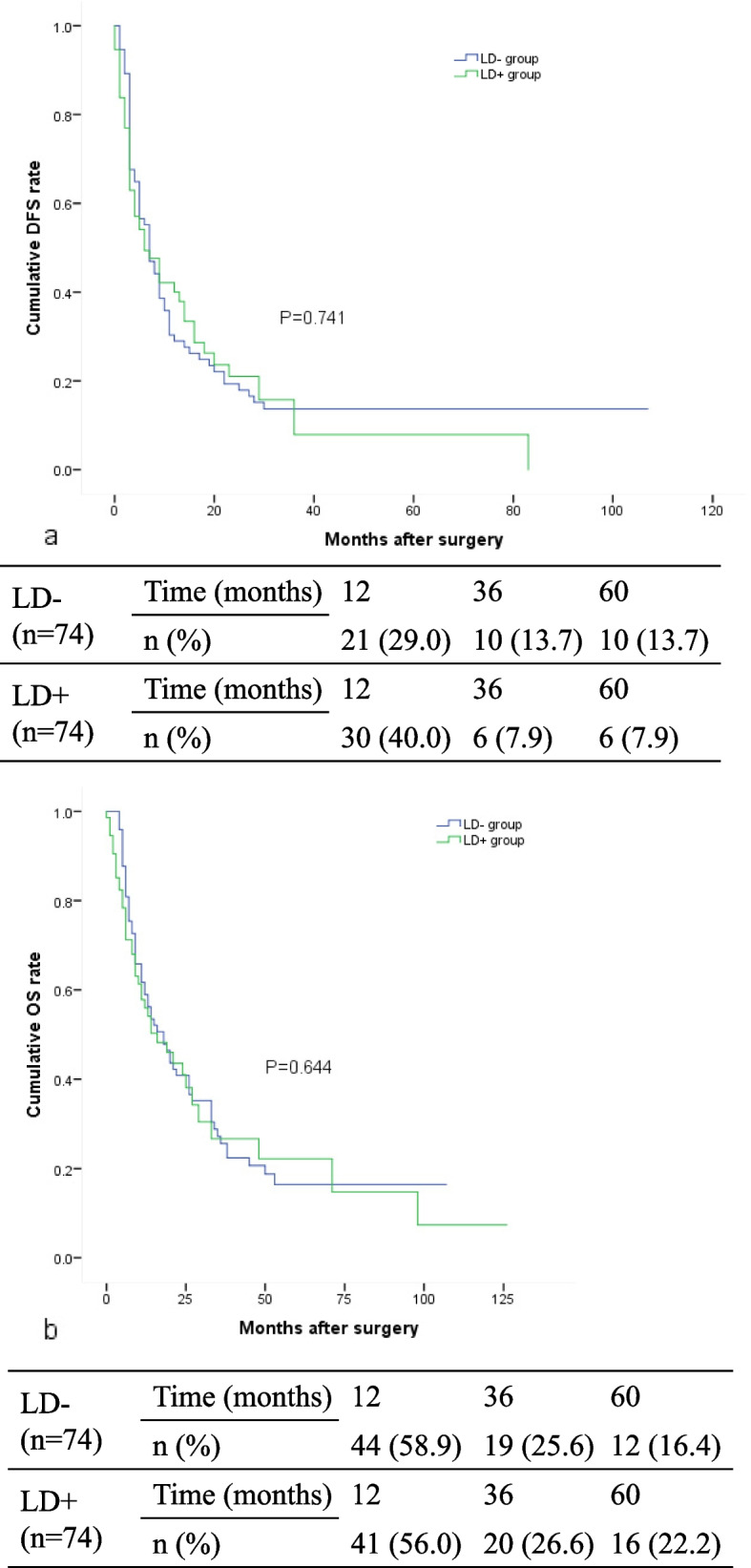
DFS rate (**a**) and OS rate (**b**) of in 74 Pairs of Matched ICC Patients after surgery according to 1:1 PSM between LD- and LD+ Patients

To investigate the association of nodal status with DFS and OS, a 1:1 PSM analysis was conducted comparing the crude LD- (*n* = 194) and LD+ (*n* = 75) groups. After matching, no significant differences were observed concerning major complications (*p* = 0.347), blood loss (*p* > 0.99) or blood transfusions (*p* > 0.99) between these two groups (Table 3). Additionally, there were no significant differences in the 1-, 3- and 5-year DFS rates (LD- vs. LD+; 29.0, 13.7 and 13.7% vs. 40.0, 7.9 and 7.9%, *p* = 0.741; Fig. 2a) or the 1-, 3- and 5-year OS rates (LD- vs. LD+; 58.9, 25.6, and 16.4% vs. 56.0, 26.6 and 22.2%, *p* = 0.644; Fig. 2b between two groups).

**Table 3:** PSM between LD- and LD+ Patients Resulting in 74 Pairs of Matched Patients

Variables	Before PSM			After PSM		
	LD(−) group *n* = 194	LD(+) group *n* = 75	*P* value	LD(−) group *n* = 74	LD(+) group *n* = 74	*P* value
Age, median (range)	54 (27–87)	56 (36–77)	0.694	53 (31–73)	56 (36–77)	0.192
Gender (male), n (%)	121 (62.4)	48 (64.0)	0.804	47 (63.5)	48 (64.9)	0.864
HBsAg positive, n (%)	107 (55.2)	41 (54.7)	0.942	44 (59.5)	40 (54.1)	0.507
Child-Pugh Class (B), n (%)	10 (5.2)	5 (6.7)	0.851	5 (6.8)	5 (6.8)	> 0.99
AST, IU/L, median (range)	31 (10–701)	31 (10–451)	0464	30 (14–701)	31 (10–451)	0.681
CA-199 (U/mL), median (range)	35.4 (0–1000)	43.8 (0–5533)	0.102	33.4 (0–1000)	48.1 (0–5533)	0.064
Tumor diameter (cm), median (range)	6 (0.5–18.4)	6.7 (2–17)	0.034	6 (0.5–15)	6.7 (2–17)	0.687
Unifocal lesions, n (%)	121 (62.4)	40 (53.3)	0.175	45 (60.8)	40 (54.1)	0.406
Major hepatectomy, n (%)	116 (59.8)	55 (73.3)	0.039	50 (67.6)	54 (73.0)	0.472
Negative surgical margin, n (%)	182 (93.8)	70 (93.3)	> 0.99	69 (93.2)	69 (93.2)	> 0.99
Blood loss (mL), median (range)	300 (50–4000)	300 (20–1500)	0.064	300 (100–4000)	300 (20–1500)	> 0.99
Transfusion, n (%)	28 (14.4)	20 (26.7)	0.019	16 (21.6)	20 (27.0)	> 0.99
Major complications, n (%)	14 (7.2)	4 (5.3)	0.579	7 (9.5)	4 (5.4)	0.347
Tumor differentiation, poor, n (%)	59 (30.4)	24 (32.0)	0.800	18 (24.3)	23 (31.1)	0.358
Macrovascular invasion, n (%)	21 (10.8)	13 (17.3)	0.150	11 (14.9)	12 (16.2)	0.821
Microvascular invasion, n (%)	34 (17.5)	15 (20.0)	0.637	13 (17.6)	15 (20.3)	0.675
Perineural invasion, n (%)	16 (8.2)	5 (6.7)	0.665	2 (2.7)	5 (6.8)	0.442
Periductal invasion, n (%)	12 (6.2)	4 (5.3)	> 0.99	2 (2.7)	4 (5.4)	0.681
Cirrhosis, n (%)	110 (56.7)	36 (48.0)	0.199	45 (60.8)	36 (48.6)	0.137
Antiviral therapy, n (%)	53 (27.3)	17 (22.7)	0.435	14 (18.9)	17 (23)	0.545
Adjuvant therapy, n (%)	67 (34.5)	30 (40.0)	0.403	35 (47.3)	29 (39.92)	0.319

*PSM* propensity score matching, *LD* lymphadenectomy, *AST* alanine aminotransferase, *CA19–9* carbohydrate antigen 19–9, *HBsAg* hepatitis B virus surface antigen

Among the LD+ patients, no significant differences in clinical features were observed between Groups N0 and N1 patients (Supplemental Table 1). Notably, the two groups displayed no significant differences in the 1-, 3- or 5-year DFS rates (N0 vs. N1; 45.4, 12.1 and 12.1% vs. 33.4%, 0, 0, *p* = 0.058, Supplemental Fig. 1a) yet there were significant differences in the 1-, 3- and 5-year OS rates (N0 vs. N1; 73.5, 39.7 and 29.8% vs. 34.9, 12.2 and 12.2%, *p* < 0.001, Supplemental Fig. 1b). Furthermore, Groups N0 patients and Group Nx patients showed no significant differences in clinical features (Supplemental Table 2) and 1-, 3- or 5-year DFS rates (N0 vs. Nx; 45.4, 12.1 and 12.1% vs. 34.0%, 20.0, 18.7, *p* = 0.780, Supplemental Fig. 2a), as well as in the 1-, 3- and 5-year OS rates (N0 vs. Nx; 73.5, 39.7 and 29.8% vs. 58.4, 31.7 and 21.0%, *p* = 0.142, Supplemental Fig. 2b). After PSM, 32 N1 patients and 32 Nx patients were compared (Supplemental Table 3), Similarly, revealing no significant differences in the 1-, 3- or 5-year DFS rates (N1 vs. Nx; 35.7%, 0 and 0 vs. 34.4, 9.4 and 9.4%, *p* = 0.249, Supplemental Fig. 3a) or in the 1-, 3- and 5-year OS rates (N1 vs. Nx; 39.5, 13.8 and 13.8% vs. 46.9, 19.5 and 7.8%, *p* = 0.195, Supplemental Fig. 3b) between these two groups.

**Fig. 3:**
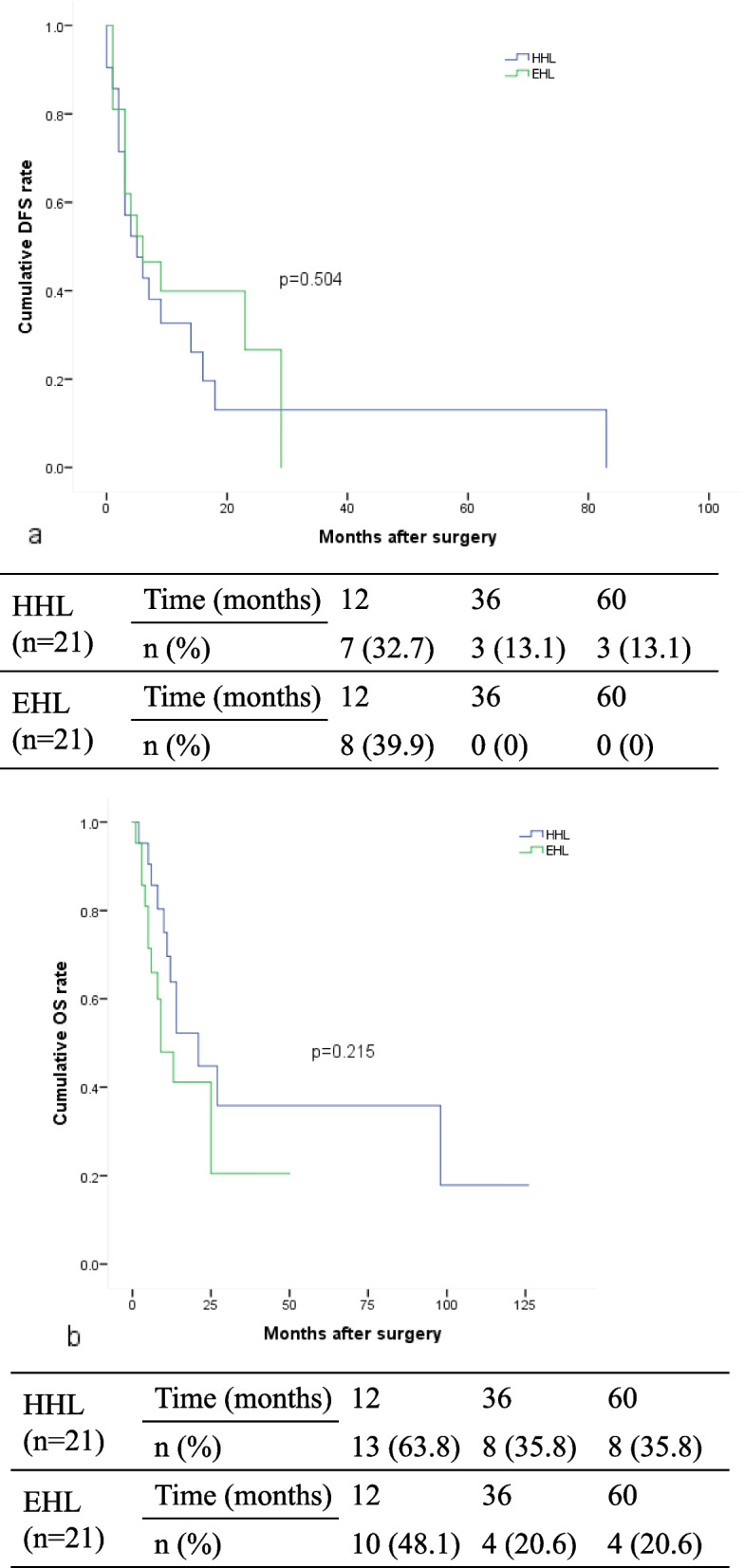
DFS rate (**a**) and OS rate (**b**) of in 21 Pairs of Matched ICC Patients after surgery according to 1:1 PSM between HHL and EHL Patients

In this study, 75 (27.9%) patients in this study underwent concomitant LD, with 48 (64%) patients receiving HHL and 27 (36%) patients undergoing EHL. Additionally, metastatic nodal disease was observed in 36 (48%) patients; the most common metastatic site was the hepatoduodenal ligament (*n* = 18, 50.0%), with some patients (*n* = 12, 33.3%) encountering the first metastatic lymph node more distantly, skipping local nodes (Table 4). The crude analysis indicated that more patients in the EHL group (*n* = 18, 66.7%) had positive lymph node metastasis than those in the HHL group (*n* = 18, 37.5%, *p* = 0.015). However, after PSM analysis, the difference in positive lymph node metastasis between the EHL and HHL groups was not significant (61.9% vs. 38.1%, *p* = 0.123). Additionally, more patients in the HHL group received transfusions than those in the EHL group (42.9% vs. 12.3%, *p* = 0.04, Table 5) after matching. No significant differences were found in the 1-, 3- and 5-year DFS rates (HHL vs. EHL; 32.7, 13.1 and 13.1% vs. 39.9%, 0 and 0, *p* = 0.504, Fig. 3a) or the 1-, 3- and 5-year OS rates (HHL vs. EHL; 63.8, 35.8 and 35.8% vs. 48.1, 20.6 and 20.6%, *p* = 0.215, Fig. 3b) between the HHL and EHL groups after matching.

**Table 4:** Sites of metastatic nodes of 36 patients with metastatic nodal disease

Sites of metastatic notes	Patients (*n* = 36)	Percentage (%)
Hepatoduodenal ligament	18	50
Peri-pancreatic	6	16.7
Distant	12	33.3

**Table 5:** PSM between HHL and EHL Patients Resulting in 21 Pairs of Matched Patients

Variables	Before PSM			After PSM		
	HHL group *n* = 48	EHL group *n* = 27	*P* value	HHL group *n* = 21	EHL group *n* = 21	*P* value
Age, median (range)	54 (36–77)	59 (37–73)	0.282	60 (39–72)	56 (337–73)	0.419
Gender (male), n (%)	32 (66.7)	16 (59.3)	0.521	10 (47.6)	12 (57.1)	0.537
HBsAg positive, n (%)	30 (62.5)	11 (40.7)	0.069	8 (38.1)	10 (47.6)	0.533
Child-Pugh Class (B), n (%)	2 (4.2)	3 (11.1)	0.344	1 (4.8)	2 (9.5)	> 0.99
AST, IU/L, median (range)	30.5 (10–449)	33 (18–451)	0.191	28 (17–154)	33 (18–451)	0.150
CA-199 (U/mL), median (range)	36.2 (0.6–5533)	80.8 (0–1000)	0.971	36.2 (0.6–5533)	123.2 (0–1000)	0.595
Tumor diameter (cm), median (range)	6.55 (3–17)	7 (2–13)	0.948	6.5 (3–17)	7 (2–13)	0.854
Unifocal lesions, n (%)	24 (50.0)	16 (59.3)	0.440	11 (52.4)	10 (47.6)	0.758
Major hepatectomy, n (%)	35 (72.9)	20 (74.1)	0.913	14 (66.7)	15 (71.4)	0.739
Negative surgical margin, n (%)	45 (93.8)	25 (92.6)	> 0.99	19 (90.5)	10 (90.5)	> 0.99
Blood loss (mL), median (range)	375 (20–1500)	300 (50–1000)	0.067	400 (20–1500)	300 (50–1000)	0.190
Transfusion, n (%)	17 (35.4)	3 (11.1)	0.022	9 (42.9)	3 (14.3)	0.040
Major complications, n (%)	3 (6.3)	1 (3.7)	> 0.99	0 (0)	1 (4.8)	> 0.99
Tumor differentiation, poor, n (%)	16 (33.3)	8 (29.6)	0.741	4 (19.0)	7 (33.3)	0.292
Macrovascular invasion, n (%)	10 (20.8)	3 (11.1)	0.453	2 (9.5)	3 (14.3)	> 0.99
Microvascular invasion, n (%)	11 (22.9)	4 (14.8)	0.400	3 (14.3)	4 (19.0)	> 0.99
Perineural invasion, n (%)	3 (6.3)	2 (7.4)	> 0.99	1 (4.8)	2 (9.5)	> 0.99
Periductal invasion, n (%)	2 (4.2)	2 (7.4)	0.616	2 (9.5)	2 (9.5)	> 0.99
Cirrhosis, n (%)	25 (52.1)	11 (40.7)	0.345	7 (33.3)	10 (47.6)	0.346
Lymph node metastasis, n (%)	18 (37.5)	18 (66.7)	0.015	8 (38.1)	13 (61.9)	0.123
Antiviral therapy, n (%)	14 (29.2)	3 (11.1)	0.073	1 (4.8)	3 (14.3)	0.606
Adjuvant therapy, n (%)	22 (45.8)	8 (29.6)	0.169	8 (38.1)	8 (38.1)	> 0.99

*PSM* propensity score matching, *HHL* hepatic hilar lymphadenectomy, *EHL* extended hepatic hilar lymphadenectomy, *AST* alanine aminotransferase, *CA19–9* carbohydrate antigen 19–9, *HBsAg* hepatitis B virus surface antigen

## Discussion

The prognosis after surgical resection of ICC remains poor because of aggressive local invasion and frequent metastasis, which tend to spread through the lymphatic system [15, 16]. Although the importance of lymph node sampling during curative cancer resection has been well established for some gastrointestinal malignancies, its role in hepatobiliary tumors is less defined [17, 18]. For instance, LD is performed for gallbladder cancer [19] and fibrolamellar hepatocellular carcinoma [20], but its role in ICC remains largely undetermined. Consequently, a wide variation in the utilization of LD in ICC has been noted globally [21–23], with LD utilization reported to range from approximately 50% in Western centers to over 75–80% in Eastern centers, with a lymph node metastasis incidence of approximately 30% in most studies [3, 4, 7, 24].

A previous study has questioned the value of LD for benefiting patient survival, arguing that routine concomitant lymph node evaluation (LNE) might be unnecessary [7]. However, several recent studies have emphasized the significance of LNE in ICC surgical management, particularly in evaluating adequate lymphadenectomy (at least 6 lymph nodes) [5, 6, 21]. Surprisingly, there were no significant differences in OS and DFS between the LD+ and LD- groups in the crude data and after PSM. We suggested that the negative effect of LD might result from the overall poor survival rates of the ICC population. Since less than one quarter of ICC patients underwent concomitant LD, the lymph node status of LD- group patients with Nx was histologically unclear. Additionally, 44.8% of patients with preoperative clinically node-negative ICC have been proven positive after adequate LD [8]. Thus, more aggressive and accurate perioperative LNE might be more crucial and practical [15, 20].

The current study demonstrated that OS was similar between Nx and N1 patients, and between Nx and N0 patients. However, OS was worse among N1 patients than among N0 patients. Moreover, multivariate analysis showed that LD was associated with improved OS. As a result, we could infer that the Nx patient group had a near 1:1 ratio of N0:N1 patients [8]. Furthermore, he study’s data suggestted that the Nx patient group might consist of a combination of N0 and understaged N1 patients, which aligns with a study by Bagante et al. [25]. However, this hypothesis of heterogeneous outcome of Nx patients could not be proven by retrospective analysis. As such, a prospective trial of routine LD is warranted. Additionally, this study suggested that routine LD would not increase the blood loss volumes, number of transfusions or postoperative complication rates, and may even improve survival, as reported previously [26].

This study has several limitations. Firstly, its retrospective nature is the main limitation. Selection bias likely impacted treatment choices. Specifically, patients with suspicious lymph nodes may have been more likely to undergo LD. However, preferential use of LD in patients with suspicious nodes would have, if anything, further underestimated the prevalence of lymph node metastasis in the Nx group. Secondly, the number of patients undergoing LD (*n* = 75, 27.8%) and N+ (*n* = 36, 13.3%) is exceptionally limited, especially after PSM (LD+ group, *n* = 74; N1 group, *n* = 32), which may account for the null significance between groups. This limitation might arise from the long duration of this study and the insufficient routine lymph node evaluation before the publication of the 8th edition of the AJCC staging manual recommendations. Consequently, these primary limitations could have biased the results toward the null hypothesis. Therefore, a multi-center, prospective and multidisciplinary clinical research is needed in the future.

## Conclusions

In conclusion, survival remains poor among patients undergoing liver resection for ICC. Lymph node metastasis is one of the strongest predictors of survival, thus guiding the prognosis and adjuvant therapy administration. Additionally, routine LD in the form of EHL would not compromise short- or long-term survival, and survival can be further improved by adjuvant therapy. Thus, LD could help identify the nodular status and potentially benefit survival, and consequently, it should be recommended as routine procedure for patients undergoing resection for ICC to achieve accurate staging and provide evidence for administering appropriate adjuvant therapy.

### Supplementary Information

**Additional file 1 : Supplemental Fig. 1.** DFS rate (a) and OS rate (b) of in 75 ICC Patients after surgery between N0 and N1 patients.

**Additional file 2 : Supplemental Fig. 2.** DFS rate (a) and OS rate (b) of in 233 ICC Patients after surgery between N0 and Nx patients.

**Additional file 3 : Supplemental Fig. 3.** DFS rate (a) and OS rate (b) of in 32 Pairs of Matched ICC Patients after surgery according to 1:1 PSM between N1 and Nx Patients.

**Additional file 4 : Supplemental Table 1.** Clinicopathological features between N0 and N1 patients.

**Additional file 5 : Supplemental Table 2.** Clinicopathological features between N0 and Nx patients.

**Additional file 6 Supplemental Table 3.** PSM between N1 and Nx Patients Resulting in 32 Pairs of Matched Patients.

## Data Availability

All data generated or analysed during this study are included in this published article [and its supplementary information files].

## Abbreviations

ICC: Intrahepatic cholangiocarcinoma
OS: Overall survival
DFS: Disease free survival
LD: Lymphadenectomy
AJCC: American Joint Committee on Cancer
AST: Aspartate aminotransferase
HBsAg: Hepatitis B virus surface antigen
MODS: Multiple organ dysfunction syndrome
IQR: Interquartile range
PSM: Propensity score-match
HHL: Hepatic hilar lymphadenectomy
EHL: Extended hepatic hilar lymphadenectomy
HCC: Hepatocellular carcinoma
