# Supplementary material for: Should lymphadenectomy performed routinely in patients with primary intrahepatic cholangiocarcinoma undergoing curative hepatectomy? A retrospective cohort study with propensity-score matching analysis
Source: BMC Surg. 2023 Nov 30;23:364. doi: 10.1186/s12893-023-02255-5 (PMC10688469; doi:10.1186/s12893-023-02255-5)
Supplement: Supplementary file 1 — Additional file 1 : Supplemental Fig. 1. DFS rate (a) and OS rate (b) of in 75 ICC Patients after surgery between N0 and N1 patients. [file 12893_2023_2255_MOESM1_ESM.ppt]

## Slide 1
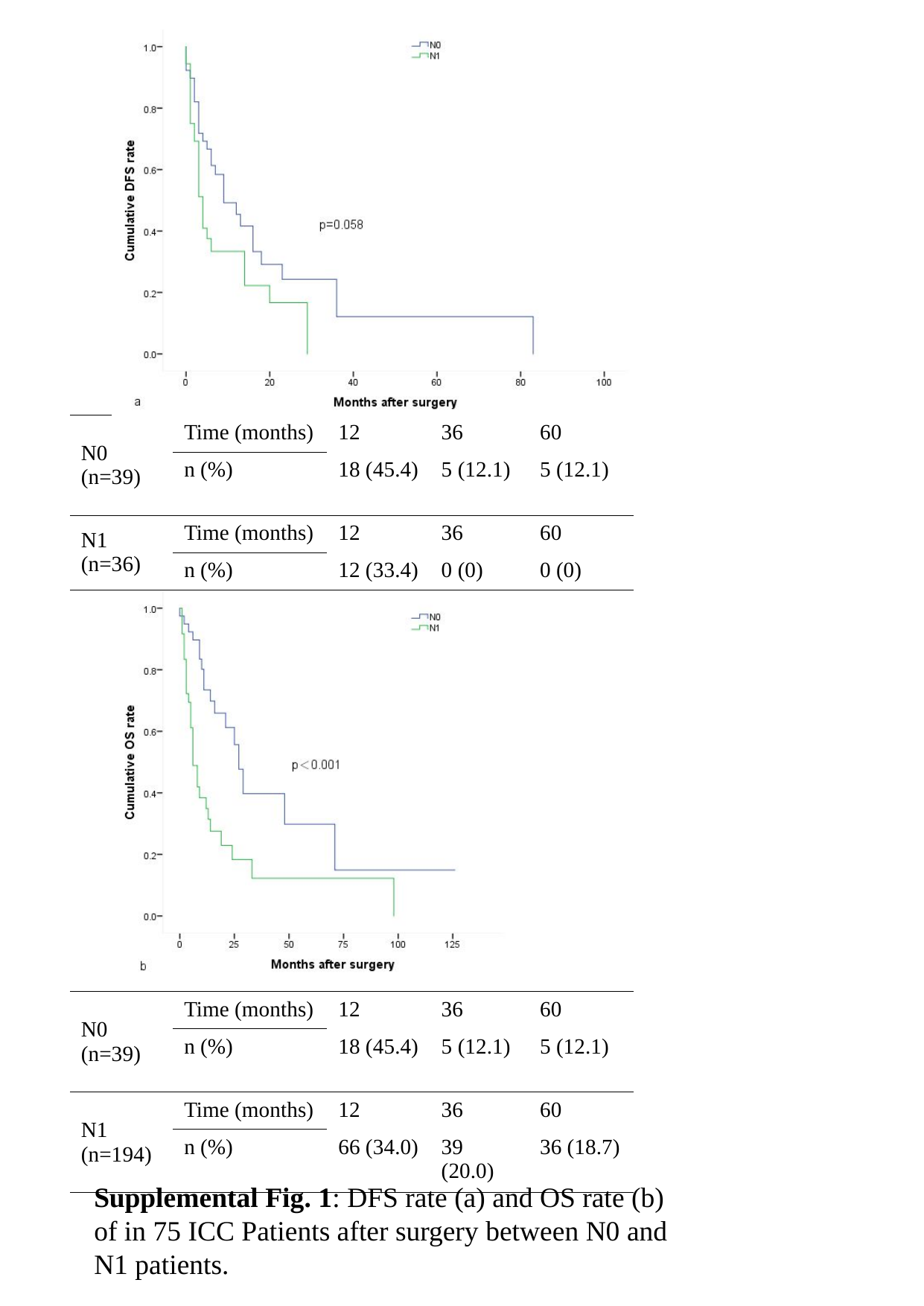

| N0 (n=39) | Time (months) | 12 | 36 | 60 |
| --- | --- | --- | --- | --- |
| | n (%) | 18 (45.4) | 5 (12.1) | 5 (12.1) |
| N1 (n=36) | Time (months) | 12 | 36 | 60 |
| | n (%) | 12 (33.4) | 0 (0) | 0 (0) |
| N0 (n=39) | Time (months) | 12 | 36 | 60 |
| --- | --- | --- | --- | --- |
| | n (%) | 18 (45.4) | 5 (12.1) | 5 (12.1) |
| N1 (n=194) | Time (months) | 12 | 36 | 60 |
| | n (%) | 66 (34.0) | 39 (20.0) | 36 (18.7) |
Supplemental Fig. 1: DFS rate (a) and OS rate (b) of in 75 ICC Patients after surgery between N0 and N1 patients.
