# Supplementary material for: Should lymphadenectomy performed routinely in patients with primary intrahepatic cholangiocarcinoma undergoing curative hepatectomy? A retrospective cohort study with propensity-score matching analysis
Source: BMC Surg. 2023 Nov 30;23:364. doi: 10.1186/s12893-023-02255-5 (PMC10688469; doi:10.1186/s12893-023-02255-5)
Supplement: Supplementary file 3 — Additional file 3 : Supplemental Fig. 3. DFS rate (a) and OS rate (b) of in 32 Pairs of Matched ICC Patients after surgery according to 1:1 PSM between N1 and Nx Patients. [file 12893_2023_2255_MOESM3_ESM.ppt]

## Slide 1
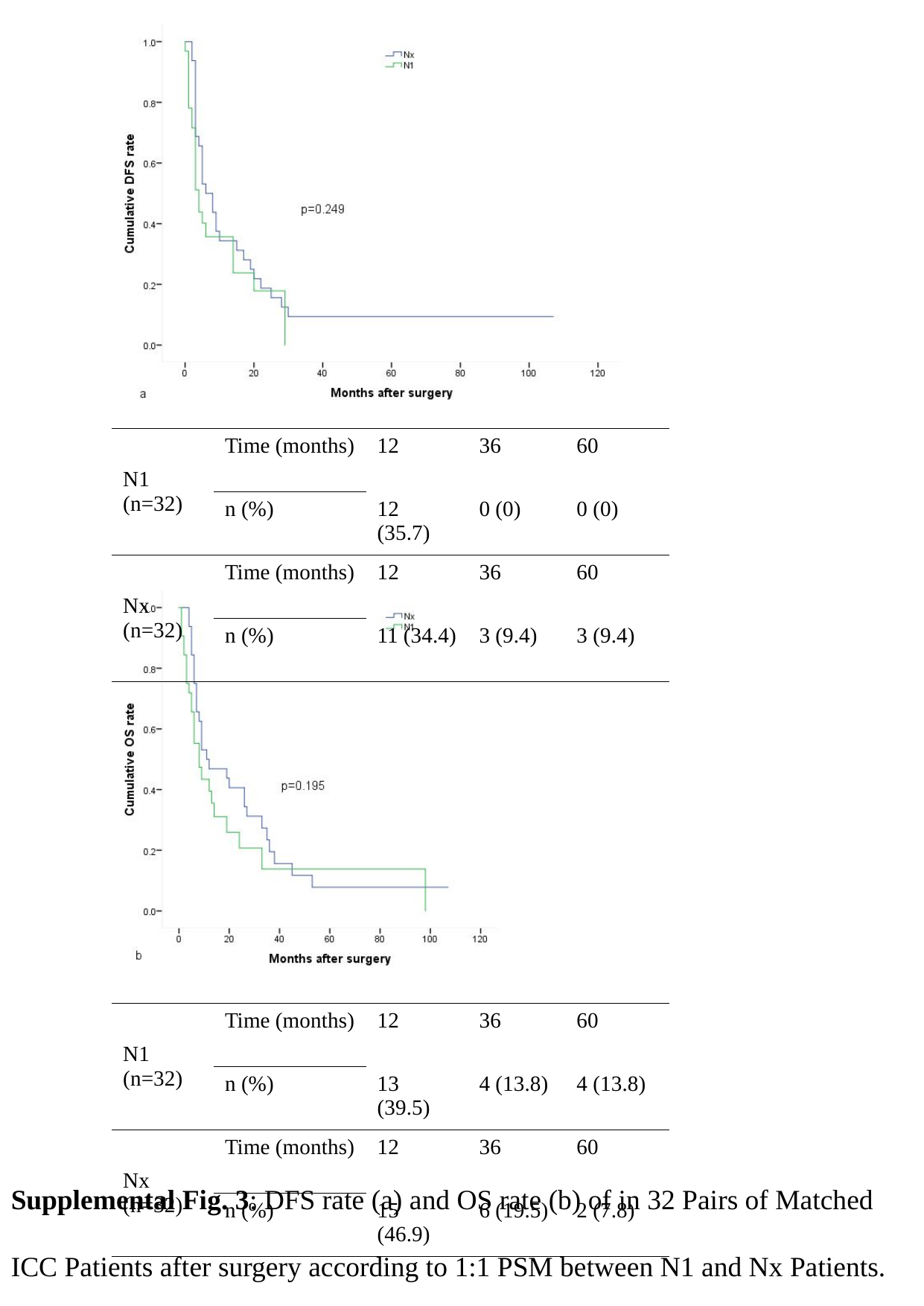

| N1 (n=32) | Time (months) | 12 | 36 | 60 |
| --- | --- | --- | --- | --- |
| | n (%) | 12 (35.7) | 0 (0) | 0 (0) |
| Nx (n=32) | Time (months) | 12 | 36 | 60 |
| | n (%) | 11 (34.4) | 3 (9.4) | 3 (9.4) |
| N1 (n=32) | Time (months) | 12 | 36 | 60 |
| --- | --- | --- | --- | --- |
| | n (%) | 13 (39.5) | 4 (13.8) | 4 (13.8) |
| Nx (n=32) | Time (months) | 12 | 36 | 60 |
| | n (%) | 15 (46.9) | 6 (19.5) | 2 (7.8) |
Supplemental Fig. 3: DFS rate (a) and OS rate (b) of in 32 Pairs of Matched ICC Patients after surgery according to 1:1 PSM between N1 and Nx Patients.
