# Supplementary material for: Should lymphadenectomy performed routinely in patients with primary intrahepatic cholangiocarcinoma undergoing curative hepatectomy? A retrospective cohort study with propensity-score matching analysis
Source: BMC Surg. 2023 Nov 30;23:364. doi: 10.1186/s12893-023-02255-5 (PMC10688469; doi:10.1186/s12893-023-02255-5)
Supplement: Supplementary file 4 — Additional file 4 : Supplemental Table 1. Clinicopathological features between N0 and N1 patients. [file 12893_2023_2255_MOESM4_ESM.docx]

| Variables | N0 group  n=39 | N1 group  n=36 | P-value |
| --- | --- | --- | --- |
| Sex, male, n (%) | 26 (66.7) | 22 (61.1) | 0.617 |
| Age, median (range, yr) | 54 (40-77) | 57 (36-72) | 0.843 |
| HBsAg, n (%) | 23 (59.0) | 18 (50.0) | 0.435 |
| Child-Pugh class B, n (%) | 2 (5.1) | 3 (8.3) | 0.666 |
| AST,IU/L, median (range) | 29 (0-449) | 34 (17-451) | 0.805 |
| CA19-9, U/mL, median (range) | 53.1 (1.5-5533) | 36.2 (0-1000) | 0.901 |
| Tumour size, cm, median (range) | 6.4 (3-13) | 7 (2-17) | 0.446 |
| Lesion, Unifocal, n (%) | 22 (56.4) | 18 (50.0) | 0.578 |
| Surgical procejure,major, n(%) | 29 (74.4) | 26 (72.2) | 0.834 |
| Negative surgical margin, n (%) | 37 (94.9) | 33 (91.7) | 0.666 |
| Blood loss (mL), median (range) | 300 (50-1500) | 300 (20-1500) | 0.758 |
| Transfusion, n (%) | 13 (33.3) | 7 (19.4) | 0.174 |
| Major complications, n (%) | 3 (7.7) | 1 (2.8) | 0.616 |
| Tumour differentiation, poor, n (%) | 9 (23.1) | 15 (41.7) | 0.085 |
| Macrovascular invasion, n (%) | 8 (20.5) | 5 (13.9) | 0.449 |
| Microvascular invasion, n (%) | 8 (20.5) | 7 (19.4) | 0.908 |
| Perineural invasion, n (%) | 1 (2.6) | 4 (11.1) | 0.188 |
| Periductal invasion, n (%) | 2 (5.1) | 2 (5.6) | >0.99 |
| Cirrhosis, n (%) | 20 (51.3) | 16 (44.4) | 0.554 |
| Antiviral therapy, n(%) | 9 (23.1) | 8 (22.2) | 0.930 |
| Adjuvant theory, n(%) | 16 (41.0) | 14 (38.9) | 0.850 |

**Supplemental Table 1.** Clinicopathological features between N0 and N1 patients

AST, alanine aminotransferase; CA19-9, carbohydrate antigen 19-9; CEA, carcinoembryonic antigen; HBsAg, hepatitis B virus surface antigen.
