# Supplementary material for: Should lymphadenectomy performed routinely in patients with primary intrahepatic cholangiocarcinoma undergoing curative hepatectomy? A retrospective cohort study with propensity-score matching analysis
Source: BMC Surg. 2023 Nov 30;23:364. doi: 10.1186/s12893-023-02255-5 (PMC10688469; doi:10.1186/s12893-023-02255-5)
Supplement: Supplementary file 5 — Additional file 5 : Supplemental Table 2. Clinicopathological features between N0 and Nx patients. [file 12893_2023_2255_MOESM5_ESM.docx]

**Supplemental Table 2.** Clinicopathological features between N0 and Nx patients.

| Variables | N0 group  n=39 | Nx group  n=194 | P-value |
| --- | --- | --- | --- |
| Sex, male, n (%) | 26 (66.7) | 121 (62.4) | 0.612 |
| Age, median (range, yr) | 54 (40-77) | 54 (27-87) | 0.849 |
| HBsAg, n (%) | 23 (59.0) | 107 (55.2) | 0.661 |
| Child-Pugh class B, n (%) | 2 (5.1) | 10 (5.2) | >0.99 |
| AST,IU/L, median (range) | 29 (0 - 449) | 31 (10-701) | 0.412 |
| CA19-9, U/mL, median (range) | 53.1 (1.5-5533) | 35.4 (0-1000) | 0.315 |
| Tumour size, cm, median (range) | 6.4 (3-13) | 6 (0.5-18.4) | 0.237 |
| Lesion, Unifocal, n (%) | 22 (56.4) | 121 (62.4) | 0.485 |
| Surgical procejure,major, n(%) | 29 (74.4) | 116 (59.8) | 0.087 |
| Negative surgical margin, n (%) | 37 (94.9) | 182 (93.8) | >0.99 |
| Blood loss (mL), median (range) | 300 (50-1500) | 300 (50-4000) | 0.205 |
| Transfusion, n (%) | 13 (33.3) | 28 (14.4) | 0.005* |
| Major complications, n (%) | 3 (7.7) | 14 (7.2) | >0.99 |
| Tumour differentiation, poor, n (%) | 9 (23.1) | 59 (30.4) | 0.358 |
| Macrovascular invasion, n (%) | 8 (20.5) | 21 (10.8) | 0.160 |
| Microvascular invasion, n (%) | 8 (20.5) | 34 (17.5) | 0.658 |
| Perineural invasion, n (%) | 1 (2.6) | 16 (8.2) | 0.319 |
| Periductal invasion, n (%) | 2 (5.1) | 12 (6.2) | >0.99 |
| Cirrhosis, n (%) | 20 (51.3) | 110 (56.7) | 0.534 |
| Antiviral therapy, n(%) | 9 (23.1) | 53 (27.3) | 0.584 |
| Adjuvant theory, n(%) | 16 (41.0) | 67 (34.5) | 0.440 |

* Indicates statistically significant.

AST, alanine aminotransferase; CA19-9, carbohydrate antigen 19-9; CEA, carcinoembryonic antigen; HBsAg, hepatitis B virus surface antigen.
