# Supplementary material for: Should lymphadenectomy performed routinely in patients with primary intrahepatic cholangiocarcinoma undergoing curative hepatectomy? A retrospective cohort study with propensity-score matching analysis
Source: BMC Surg. 2023 Nov 30;23:364. doi: 10.1186/s12893-023-02255-5 (PMC10688469; doi:10.1186/s12893-023-02255-5)
Supplement: Supplementary file 6 — Additional file 6 Supplemental Table 3. PSM between N1 and Nx Patients Resulting in 32 Pairs of Matched Patients. [file 12893_2023_2255_MOESM6_ESM.docx]

**Supplemental Table 3.** PSM between N1 and Nx Patients Resulting in 32 Pairs of Matched Patients.

| Variables | Before PSM | | |  |  | After PSM | |
| --- | --- | --- | --- | --- | --- | --- | --- |
|  | N1 group  n = 36 | Nx group  n = 194 | *P* value |  | N1 group  n = 32 | Nx group  n = 32 | *P* value |
| Age, median (range) | 22 (61.1) | 121 (62.4) | 0.886 |  | 20 (62.5) | 19 (59.4) | 0.798 |
| Gender (male), n (%) | 57 (36-72) | 54 (27-87) | 0.686 |  | 57.5 (36-72) | 53.5 (31-72) | 0.388 |
| HBsAg positive, n (%) | 18 (50.0) | 107 (55.2) | 0.569 |  | 16 (50.0) | 18 (56.3) | 0.616 |
| Child-Pugh Class (B), n (%) | 3 (8.3) | 10 (5.2) | 0.434 |  | 3 (9.4) | 1 (3.1) | 0.613 |
| AST, IU/L, median (range) | 34 (17 - 451) | 31 (10-701) | 0.663 |  | 33.5 (17-451) | 31 (14-262) | 0.444 |
| CA-199 (U/mL), median (range) | 36.2 (0-1000) | 35.4 (0-1000) | 0.077 |  | 36.2 (0-1000) | 34.5 (0-1000) | 0.152 |
| Tumor diameter (cm), median (range) | 7 (2-17) | 6 (0.5-18.4) | 0.041* |  | 7 (2-17) | 6.5 (2-15) | 0.967 |
| Unifocal lesions, n (%) | 18 (50.0) | 121 (62.4) | 0.163 |  | 18 (56.3) | 21 (65.6) | 0.442 |
| Major hepatectomy, n (%) | 26 (72.2) | 116 (59.8) | 0.159 |  | 22 (68.8) | 22 (68.8) | >0.99 |
| Negative surgical margin, n (%) | 33 (91.7) | 182 (93.8) | 0.711 |  | 30 (93.8) | 32 (100) | 0.492 |
| Blood loss (mL), median (range) | 300 (20-1500) | 300 (50-4000) | 0.140 |  | 300 (20-1500) | 300 (100-4000) | 0.911 |
| Transfusion, n (%) | 7 (19.4) | 28 (14.4) | 0.442 |  | 5 (15.6) | 8 (25) | 0.351 |
| Major complications, n (%) | 1 (2.8) | 14 (7.2) | 0.477 |  | 0 (0) | 4 (12.5) | 0.113 |
| Tumor differentiation, poor, n (%) | 15 (41.7) | 59 (30.4) | 0.184 |  | 11 (34.4) | 9 (28.1) | 0.590 |
| Macrovascular invasion, n (%) | 5 (13.9) | 21 (10.8) | 0.805 |  | 3 (9.4) | 4 (12.5) | >0.99 |
| Microvascular invasion, n (%) | 7 (19.4) | 34 (17.5) | 0.782 |  | 6 (18.8) | 5 (15.6) | 0.740 |
| Perineural invasion, n (%) | 4 (11.1) | 16 (8.2) | 0.528 |  | 2 (6.3) | 2 (6.3) | >0.99 |
| Periductal invasion, n (%) | 2 (5.6) | 12 (6.2) | >0.99 |  | 2 (6.3) | 0 (0) | 0.492 |
| Cirrhosis, n (%) | 16 (44.4) | 110 (56.7) | 0.175 |  | 16 (50.0) | 17 (53.1) | 0.802 |
| Antiviral therapy, n (%) | 8 (22.2) | 53 (27.3) | 0.525 |  | 8 (25.0) | 4 (12.5) | 0.200 |
| Adjuvant therapy, n (%) | 14 (38.9) | 67 (34.5) | 0.616 |  | 12 (37.5) | 17 (53.1) | 0.209 |

* Indicates statistically significant.

AST, alanine aminotransferase; CA19-9, carbohydrate antigen 19-9; CEA, carcinoembryonic antigen; HBsAg, hepatitis B virus surface antigen; PSM, propensity score matching.
